# Microbial Consortia Are Needed to Degrade Soil Pollutants

**DOI:** 10.3390/microorganisms10020261

**Published:** 2022-01-24

**Authors:** Ting Zhang, Houjin Zhang

**Affiliations:** MOE Key Laboratory of Molecular Biophysics, Department of Biotechnology, College of Life Science and Technology, Huazhong University of Science and Technology, Wuhan 430074, China; t_zhang@hust.edu.cn

**Keywords:** microbial consortium, soil pollutants, biodegradation efficiency, synergistic degradation

## Abstract

Soil pollution is one of the most serious environmental problems globally due to the weak self-purification ability, long degradation time, and high cost of cleaning soil pollution. The pollutants in the soil can be transported into the human body through water or dust, causing adverse effects on human health. The latest research has shown that the clean-up of soil pollutants through microbial consortium is a very promising method. This review provides an in-depth discussion on the efficient removal, bio-adsorption, or carbonated precipitation of organic and inorganic pollutants by the microbial consortium, including PAHs, BPS, BPF, crude oil, pyrene, DBP, DOP, TPHP, PHs, butane, DON, TC, Mn, and Cd. In view of the good degradation ability of the consortium compared to single strains, six different synergistic mechanisms and corresponding microorganisms are summarized. The microbial consortium obtains such activities through enhancing synergistic degradation, reducing the accumulation of intermediate products, generating the crude enzyme, and self-regulating, etc. Furthermore, the degradation efficiency of pollutants can be greatly improved by adding chemical materials such as the surfactants Tween 20, Tween 80, and SDS. This review provides insightful information regarding the application of microbial consortia for soil pollutant removal.

## 1. Introduction

The migration of organic pollutants, such as polycyclic aromatic hydrocarbons (PAHs), degrade dibutyl phthalate (DBP), di-*n*-octyl phthalate (DOP), and volatile organic compounds (VOCs), can cause soil pollution [1]. Pollutants can be transferred to the soil indirectly from various wastes, including antibiotics for hospital medical waste, industrial wastewater, waste treatment plants, and various point sources such as landfills, container leaks, waste slag pits, and mine tailings [2,3]. Every country has thousands of sites that have been contaminated by pollutants [2]. However, due to many applications of these chemicals, developing countries such as China, India, Vietnam, Nigeria, Ethiopia, Pakistan, Indonesia, and South Africa may have more contaminated sites than developed countries [2,3]. Pollutant concentration and mutual dependence vary from country to country [2,4]. The dissolution of heavy metals can also pollute the soil. For example, Cd is a well-known heavy metal [5]. These pollutants, even at low concentrations, can cause cancer and deformities [2,6,7]. Inhalation, ingestion, or direct contact with these pollutants will adversely affect the human body, leading to human physical and mental diseases, such as respiratory diseases, cardiovascular diseases, cancers, etc. [2,7]. Humans and other creatures, including birds and amphibians, have also been negatively affected [2,4]. Pollutants in the soil, once absorbed by earthworms and then eaten by birds, accumulate in bird populations, which has been reported to be the main reason for the decline in bird populations [2]. The mortality, metabolic and reproductive damage, extinction, acute mortality, and reduced fertility of various birds have also been reported due to long-term exposure to toxic pollutants [2]. Soil pollution by organic and inorganic matter is one of the most severe environmental challenges globally, and it requires immediate, effective, and sustainable solutions [2,4]. Therefore, there is an urgent need to reduce soil pollution caused by heavy metals and organic pollutants. Technologies to remove heavy metals and organic pollutants in the soil are essential to prevent environmental and health problems [5,8].

Purifying soil contaminated by toxic pollutants is a critical task [2]. In the natural environment, harmful pollutants can be removed through biological, chemical, and photochemical degradation [3,4,9,10,11,12,13,14]. The degradation of pollutants depends on the stability of the pollutants, degradation kinetics, and physical/chemical environment [2]. Physical and chemical methods of removing pollutants include coagulation, precipitation, centrifugation, adsorption and desorption, hydrolysis, and photodegradation [2,15]. However, these processes are costly and have limited effects [2,15]. On the other hand, the biological decomposition of pollutants is a cheap and environmentally friendly method that can be used to purify contaminated soil [2,16]. Biodegradation is the catalytic reduction of pollutants mediated by microbial enzymes. Microorganisms have degrading enzymes, which cleave the original bonds of toxic pollutants and convert them into inorganic forms through mineralization [2,15]. This is a practice that uses microorganisms to convert harmful pollutants into less toxic or non-toxic forms [2]. It is generally believed that microorganism biodegradation is the primary mechanism of dissipating organic pollutants [3,4,9,10,11,12,13,14]. Moreover, compared to other methods, biodegradation is a practical and eco-friendly method, so it has been widely used [2,15]. To make full use of microbial resources, the degradation ability of microorganisms should be studied. The success of bioremediation in contaminated sites depends on the survival rate of microorganisms and their high cell density, stability, and reusability [17].

Many studies have shown that it is difficult to achieve the complete degradation of pollutants by a single strain. As different strains have different metabolic pathways, the bacteria with different removal abilities are mixed, and the microbial consortium can integrate each strain’s advantages to achieve the efficient degradation of pollutants. Mixed microbial consortia exhibited good performance in substrate tolerance and enhanced pollutant degradation [2,8,9,13,14,18,19]. Compared to the culture of a single strain, the performance of the consortium of microorganisms is better. The microbial consortium showed apparent effects in the degradation of pollutants [2]. Some existing microbial strains isolated from the intestinal flora and natural flora have the inherent ability to degrade pollutants [2,16]. *Lactobacilli*, *Actinobacteria*, *Pseudomonas*, *Clostridium*, *Salmonella*, and *Escherichia coli* have been found to have the inherent ability to degrade pollutants [2]. These strains are suitable for the bioremediation of pollutants [2,15]. The microbial consortium has become an important technology because it degrades pollutants more effectively than a single strain [2,16]. Bioremediation is usually carried out by the microbial consortium rather than by individual species in the natural environment, and different strains or species play different functional roles [17]. The co-cultivation of the microbial consortium is more effective than single bacteria, degrades pollutants faster, and can significantly enhance the biodegradation of pollutants in the soil [8,17].

The bacterial consortium can effectively bioremediate contaminated sites. The mechanism can be summarized as follows: First, the synergistic metabolic degradation of the bacterial consortium increases. The bacterial consortium members can degrade the essential intermediate compounds produced by other members in degrading pollutants and reducing the accumulation of intermediate products, thereby increasing the metabolic pathways for the biodegradation of organic pollution (Mechanism 1). Second, some bacterial consortium strains produce many high-efficiency biosurfactants, thereby increasing the solubility and content of pollutants, improving their bioavailability and biodegradability (Mechanism 2). Third, the microbial consortium can self-regulate and adapt during degradation. Microbial consortia show better performance than individual cultures in degrading contaminants (Mechanism 3). Fourth, the microbial consortium can promote the growth of strains by using metabolites after pollutant degradation (Mechanism 4). Fifth, the crude enzyme produced in the microbial consortium’s intracellular space can be used as a degradation factor in degradation, showing high degradation activity (Mechanism 5). Sixth, there is a biochemical synergistic effect between bacterial consortium strains, which enhances bacterial activity and the degradation of pollutants (Mechanism 6). Therefore, the microbial consortia show a high ability to degrade soil pollutants.

In addition, the strains and the microalgal consortium can significantly improve the degradation and adaptability of microbial cells. The addition of surfactants to a bacterial consortium has demonstrated a better ability to remove pollutants than the bacterial consortium alone. Moreover, the biochar or immobilized laccase added to the bacterial consortium may also improve the bacterial consortium’s biodegradation. Therefore, these substances can supplement the bacterial consortium to improve the ability of microorganisms to degrade pollutants.

**Figure 1:**
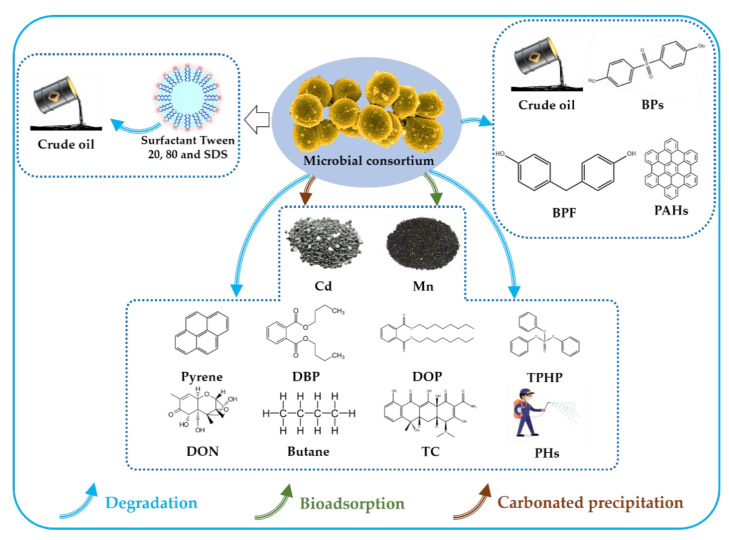
Degradation of contaminants by microbial consortia. The combination of bacteria or fungi strains efficiently removes contaminants such as PAHs, BPs, BPF, and crude oil. The co-cultivation of microbial strains can effectively clear contaminants such as pyrene, DBP, DOP, TPHP, PHs, butane, DON, and TC. The consortia can also convert Cd to carbonate precipitation and can bio-adsorb Mn. The addition of the surfactants Tween 20, Tween 80, and SDS to microbial consortia can enhance crude oil degradation.

This review describes the effective biodegradation of pollutants by a microbial consortium composed of bacteria or fungi as exemplified by the biodegradation of different pollutants (as shown in Figure 1). Furthermore, the specific microorganisms and detailed mechanisms are summarized. The microbial consortium is a feasible technology for the remediation of contaminated soil, which inspires the biodegradation and bioremediation of pollutants.

## 2. The Essential Roles of Consortia Composed of All Bacterial Strains in the Degradation of Contaminants

### 2.1. Efficient Removal of Pyrene by Bacterial Consortia

Pyrene is a kind of PAH with four aromatic rings [20]. The degradation of pyrene is initiated by hydrogen peroxide, and complete catabolism occurs through the cycles of phthalates, protocatechins, and tricarboxylic acids [20]. The pyrene-degrading microbial consortium can be used to clean up pyrene-contaminated sites [21].

It has been reported that a pyrene-degrading microbial consortium has been obtained from mangroves in Thailand. This consortium is composed of five cultivable bacteria (*Mycobacterium* spp. PO1 and PO2, *Novosphingobium pentaromativorans* PY1, *Ochrobactrum* sp. PW1, and *Bacillus* sp. FW1) [21]. Compared to a single bacterium, this microbial consortium has a higher pyrene degradation rate. The enhanced biodegradation of pyrene in this microbial consortium is due to the synergistic interaction of the bacterial mixture [21]. The main reason for this is that the members of the bacterial consortium can degrade the essential intermediate compounds produced during pyrene degradation by other members; that is, they can degrade phthalates or protocatechuates [21]. At the same time, *Bacillus* sp. FW1 produces a large amount of high-efficiency biosurfactant, thereby increasing the solubility of the pyrene content, improving its bioavailability and biodegradability. Therefore, this bacterial consortium shows a high ability to degrade pyrene [21]. Research has shown that the synergistic degradation of pyrene by pyrene-degrading microbial consortia can complete the bioremediation of pyrene-contaminated sites, which promotes the application of bacterial consortia in bioremediation [21]. Other studies have shown that bacterial consortia are effective at removing pyrene from the soil through natural and assisted dissipation, which provided a breakthrough for the sustainable application of bacterial consortia in the ecosystem [22,23,24,25,26,27,28,29].

### 2.2. Enhanced Degradation of DBP by Bacterial Consortia

DBP is a member of phthalic acid esters (PAEs) family and can be used as plasticizers for architectural decoration [30]. DBP is widely used as a plasticizer and is quickly released into various environments [31]. DBP cannot be easily removed by environmental hydrolysis and photolysis. Biodegradation is a vital method for PAE removal [32].

One study determined that a stable bacterial consortium (B1) could be obtained from activated sludge of a municipal sewage treatment plant. This bacterial consortium was composed of *Pandoraea* sp. and *Microbacterium* sp. and could efficiently degrade DBP. The degradation rate was able to exceed 92% over the course of three days. The optimal temperature for DBP degradation was 30 °C, and the bacterial consortium B1 was able to adapt to a wide range of pH values (5.5–8.5). In addition to DBP, the bacterial consortium B1 was also able to degrade dimethyl phthalate (DMP), di-2-ethylhexyl phthalate (DEHP), and phthalic acid (PA) [31]. It has been reported that the high diversity of bacterial consortia can improve the environmental adaptability and biodegradation efficiency against organic pollutants. In mixed culture, one strain of bacteria can use the intermediates produced by another strain [32]. A bacterial consortium has various metabolic capabilities, thereby increasing the metabolic pathways for the biodegradation of organic pollution [32]. Acidic intermediate PA is produced during the degradation of DBP. The accumulation of PA in the medium causes the pH to decrease, thereby inhibiting DBP degradation. The bacterial consortium B1 can degrade PA and prevent the accumulation of acidic substances [32]. The bacterial consortium B1 can degrade DBP well under acidic conditions and has the advantage of reducing the accumulation of intermediate products during the degradation of DBP, which is beneficial to the environment [32]. Compared to a single strain, adding bacterial consortium B1 to soil contaminated with DBP can significantly increase the DBP removal rate, which indicates that this bacterial consortium has great potential for the bioremediation of DBP-contaminated environments [32]. The use of a bacterial consortium is an effective way to remove DBP from the soil, providing a good framework for the bioremediation of DBP-contaminated soil [30,31,32].

### 2.3. Reinforced Degradation of DOP by Bacterial Consortia

DOP belongs to the phthalic acid ester (PAE) family and is one of the most commonly used plasticizers [33,34,35]. DOP can easily leak from products into the surrounding environment, resulting in soil contamination [35]. DOP is the endocrine-disrupting chemical (EDC), which is closely related to the progression of certain metabolic diseases, including obesity and diabetes [34]. Studies have shown that compared to photolysis and chemical methods, the metabolic decomposition of DOP by microorganisms is the primary method and has many advantages [35].

It has been reported that two strains of Arthrobacter have been isolated from activated sludge, *Arthrobacter* sp. SLG-4 and *Rhodococcus* sp. SLG-6, which use DOP as their only carbon. Both energy sources can degrade DOP. An analysis of DOP degradation intermediates showed that *Arthrobacter* sp. SLG-4 can completely degrade DOP. DOP cannot be mineralized by *Rhodococcus* sp. SLG-6, and the final metabolite is phthalic acid (PA) [35]. *Arthrobacter* sp. SLG-4 degrades DOP to convert DOP to PA through the de-esterification pathway, which is metabolized to protocatechuate acid and finally to tricarboxylic acid (TCA) through the meta-cleavage pathway. Additionally, the phthalate 3,4-dioxygenase genes (*phtA*) responsible for PA degradation in *Arthrobacter* sp. SLG-4 were successfully detected by real-time quantitative PCR (q-PCR) [35]. The co-culture of SLG-4 and SLG-6 significantly improved the degradation efficiency of DOP. By inoculating the bacterial consortium that degrades DOP, more than 91% of the DOP can be removed, effectively enhancing DOP degradation [35]. This study showed that inoculation with a bacterial consortium degrades DOP effectively, and this thus represents a feasible technology for DOP bioremediation in actual engineering [35]. Studies have shown that the bacterial consortium composed of *Ochrobactrum* sp. VA1. (which degrades PAHs), *Penicillium chrysogenum* (which degrades phenol), *Bacillus* sp. (which degrades hydrocarbons), *Sphingobium* sp. (which degrades di-n-butyl phthalate), *Lipomyces tetrasporus,* and *Paecilomyces variotii* (which degrades crude petroleum in sea water) can efficiently degrade the DOP in soil [34]. This is possible because the bacterial consortium is more resilient to adverse environments or environmental changes than single strain cultures [34]. The bacterial consortium degrades DOP over a wide range of temperatures and pH levels, and it is also able to degrade the intermediates produced during DOP degradation(a total of six major intermediates, hexyl octyl phthalate (HOP), di-hexyl phthalate (DHP), butyl octyl phthalate (BOP), butyl hexyl phthalate (BHP), di-butyl phthalate (DBP) and mono-butyl phthalate (MBP)) [34]. It has also been reported that this bacterial consortium can effectively degrade DOP in soil, indicating its potential to be used to remediate DOP-polluted sites [34].

### 2.4. Efficient Degradation of Triphenyl Phosphate (TPHP) by Bacterial Consortia

TPHP, which is often detected in various environments, such as in the air, water, and soil, has attracted widespread attention due to its adverse effects on organisms [36,37,38]. TPHP has a damaging effect on the respiratory tract [36,37,38]. TPHP induces cell apoptosis by inhibiting cell viability and has a toxic effect [36,37,38]. The microbial degradation of this chemical is an effective and environmentally compatible method that provides a viable option for remedying TPHP pollution [36].

Studies have shown that a novel microbial consortium GYY with the ability to degrade TPHP efficiently has been isolated and is composed of *Pseudarthrobacter*, *Sphingopyxis*, *Methylobacterium,* and *Pseudomonas* [36]. Under optimal conditions, 92.2% of TPHP can be degraded within 4 h [36]. Additionally, it was shown that TPHP was metabolized by hydrolysis, methoxylation after hydrolysis, and methoxylation after the activation of the hydroxylation pathways [36]. This was mainly due to the synergy between the different strains of microbial consortium GYY. Due to the synergy, the degradation rate of microbial consortium GYY was much higher than that of individual bacteria [36]. Among them, the methyltransferase produced by *Methylobacterium* promoted the production of methylated products. Moreover, the microbial consortium GYY can self-regulate and adapt during the degradation of TPHP [36]. The study showed that this microbial consortium can efficiently degrade TPHP, providing new ideas for the metabolic transformation of TPHP and providing a bioremediation technology for TPHP pollution [36,37,39].

### 2.5. Intensified Degradation of Phenylurea Herbicides (PHs) by Bacterial Consortia

PHs are usually detected as major water pollutants in areas where they are widely used [40,41,42,43]. Studies have shown that the *Diaphorobacter* sp. strain LR2014-1 and the *Achromobacter* sp. strain ANB-1 were isolated from a linuron (a selective herbicide)-mineralizing consortium [43]. The former first hydrolyzed linuron to 3,4-dichloroanaline, while the latter further mineralized the aniline derivative that was produced [41,42]. The synergistic catabolism of linuron by the consortium containing these two strains led to the more effective catabolism of linuron and, at the same time, promoted the growth of these two strains [43]. Strain LR2014-1 contains two evolutionarily different hydrolases, the amide hydrolase superfamily Phh and the amidase superfamily TccA2, which have complementary roles in the hydrolysis of different types of PHs, including N-methoxy-N-methyl-substituted, diuron, chlorotoluron, fluomethuron, N,N-dimethyl-substituted, and siduron [43]. This bacterial consortium can contain synergistic catabolic species for PH mineralization, and the strains can have functionally complementary hydrolases, thereby expanding the range of substrates [43]. The bacterial consortium can contain metabolically synergistic species for PH mineralization, which is a highly effective strategy for PH degradation [43,44,45].

### 2.6. Efficient Degradation of Butane by Bacterial Consortia

Hydrocarbon-degrading bacteria play an important role in eliminating the hydrocarbon pollution caused by leaking in the oil extraction process [46,47,48]. Therefore, discovering the syntrophic relationships between alkane and alcohol-oxidizing bacteria is of great significance for improving the bioremediation efficiency of hydrocarbon contamination [47].

The oxidation of butane by hydrocarbon-degrading bacteria has been described for a long time, but little is known about the microbial interactions in this process [46]. In a recent study on this interaction, the efficiency of butane oxidation was evaluated in a single culture and co-culture of six butane-oxidizing bacteria (BOB, PG-3-1, PG-3-6, PG-3-2, PG-3-10, PG-3-7, and PG-3-12) and the butanol-oxidizing strain *Mycobacterium* sp. PG-3-5 [47]. The results of this study show that in a co-culture of seven strains, the degradation rate of butane was at least 26 times that of the six single cultures [47]. The chromatographic analysis of the gas in the metabolites showed that butanol accumulated in a single culture of the BOB strain but not in a co-culture with a butanol-oxidizing strain [47]. These results prove a new homeotropic relationship between BOB and butanol-oxidizing bacteria during butane oxidation [47]. The BOB strain oxidized butane to butanol, but this activity was inhibited by the butanol accumulated in the single culture. The butanol-oxidizing strain removed butanol in the co-culture to eliminate the inhibition, which improved the butane degradation efficiency [47]. During co-cultivation, both the BOB and butanol-oxidizing bacteria could grow, and the time required to remove butane altogether was shortened from more than 192 h to less than 4 h [47]. The synergistic effect of the co-culture was also consistent with the reverse transcription-quantitative real-time PCR (RT-qPCR) results of the *bmoX* gene because compared to the monoculture, the expression of this gene was detected to increase during the vegetative growth period, which indicates that the nutrient interaction up-regulated *bmoX* [47]. The bacterial consortium was able to greatly improve the butane degradation efficiency and showed the great potential of this new consortium in the degradation of refractory industrial pollutants [47,49].

### 2.7. Degradation of Deoxynivalenol (DON) by Bacterial Consortia

DON is a widely distributed mycotoxin that is often found in various agricultural raw materials and feeds [50,51,52]. DON is a pathogenic factor that can accelerate the spread of plant diseases [50]. In addition, its accumulation in grains can lead to a decline in yield and can cause serious health problems for humans and livestock [50,52]. The use of naturally occurring microorganisms to biodegrade DON into less toxic or non-toxic substances is considered the best way to detoxify DON [50,51,52]. Studies have isolated the bacteria that are able to degrade DON from soil samples [52]. Using a mineral medium containing 50 μg/mL DON as the sole carbon source under aerobic conditions, 85 soil samples from different provinces in China were enriched. The bacterial consortium LZ-N1 exhibits efficient and stable DON-transforming activity [51,52]. Using high-throughput sequencing technology to analyze the bacterial colony composition, 16S rRNA sequence analysis showed that the LZ-N1 bacterial consortium was composed of at least 11 bacterial genera, among which *Pseudomonas* accounted for nearly half of the relative abundance [51,52]. Two new strains from the LZ-N1 bacterial consortium, *Pseudomonas* sp. Y1 and *Lysobacter* sp. S1, were mixed and incubated [51,52]. The results show that DON was continuously converted into metabolite 3-epi-deoxynivalenol (3-epi-DON), and no degradation products were found after 72 h [51,52]. The mixed culture of cell-free supernatant, lysate, and cell debris were all able to degrade DON. Under the action of 50 μg/mL DON, the degradation rate of DON in the supernatant was able to reach 100% within 48 h [51,52]. The primary mechanism was the bacterial consortium composed of Y1 and S1 and was able to convert DON into non-toxic 3-epi-DON. DON degradation is the process of DON epimerization. DON epimerization is a two-step enzymatic detoxification pathway of DON that is ubiquitous in soil microorganisms, including in the oxidation of DON to 3-keto-deoxynivalenol (3-keto-DON) and then the selective reduction of 3-keto-DON to 3-epi-DON. The compound 3-keto-DON is the main intermediate accumulated by 3-epi-DON in other normal strains. However, this bacterial consortium’s epimerization mechanism on DON is a two-step continuous enzymatic reaction that first converts DON to 3-keto-DON and then continuously converts 3-keto-DON to 3-epi-DON. There is no accumulation of 3-keto-DON. The synergistic metabolism of the bacterial consortium promoted DON epimerization [51,52]. The study reported that using a mixed culture method with a bacterial consortium that degrades DON is better able to degrade DON, which provides news idea for how to detoxify DON-contaminated grains and feeds in the future [50,51,52,53,54].

### 2.8. Efficient Removal of Tetracycline (TC) by Bacterial Consortia

TC, an environmental pollutant, can stay in the soil for many years and can destroy the ecosystem [55]. So far, there have been many methods that have been developed to deal with TC pollution. Microbial remediation is a method that uses microorganisms to biodegrade pollutants [55,56]. It is considered to be a cost-effective and more suitable method for soil remediation [56].

One study reported a TC-degrading bacterial consortium composed of *Raoultella* sp. XY-1 and *Pandoraea* sp. XY-2 strains that was isolated and constructed from TC-contaminated soil [56]. Compared to a single strain, this TC-degrading bacterial consortium grew better and was able to degrade TC more efficiently [56]. This is due to the presence of a biochemical synergistic effect between the bacteria, which enhanced the bacteria’s activity and the TC degradation. As the logarithmic growth phase began, the TC concentration decreased faster, which indicated that once the bacteria reached a specific number, the TC biodegradation would increase. The biochemical synergy of the bacterial combination can enhance TC biodegradation to a certain extent [56]. This bacterial consortium is able to degrade the TC in the soil environment with high efficiency and has sound ecological effects [56]. The bacterial consortium degradation method can be used to treat TC pollution, and this method is beneficial to the remediation of contaminated soil and the promotion of plant growth, providing a new way through which the bioremediation of TC pollution can be explored [55,56,57,58].

### 2.9. Efficient Carbonated Precipitation of Cadmium (Cd) by Bacterial Consortia

Compared to other heavy metals, Cd is one of the most common and dangerous environmental pollutants [59,60]. A large amount of Cd is released into the environment [60,61]. Cd contamination in grains (such as rice) has also been found in many Asian countries [5,59]. Many methods to remedy Cd-contaminated water and soil have been proposed, including adsorption, chemical precipitation, electrodeposition, membrane separation, and biological methods [59]. Among the proposed methods, biological treatments such as bioremediation or microbial-mediated remediation have received extensive attention [59]. They are relatively sustainable, environmentally friendly, and low-cost and can efficiently degrade the pollutant Cd [59].

Researchers have constructed a stable urease-producing consortium (UPC) to efficiently induce the precipitation of Cd carbonate and induce heavy metals to transform from their ionic state to their stable form, thereby reducing the mobility and toxicity of these harmful metals [59]. The bacterial consortium consisted of three bacteria belonging to the phylum *Firmicutes*. It constitutes UPC (70.22–75.41% of *Sporosarcina*, 13.83–20.66% of norank_f_*Bacillaceae*, and 5.91–13.69% of unclassified_f_*Bacillaceae*) [59]. UPC has an excellent ability to convert Cd^2+^ into carbonate precipitation under various environmental conditions (pH range of 4.0–11.0, and a temperature range of 10–45 °C) [59]. The main mechanism consists of UPC containing a bacterial consortium with high urease activity and carbonate formation ability [59]. In the urease hydrolysis process of urea, bacterial cells adsorb Cd^2+^ cations through their negative charges, and the combination of Cd^2+^ and carbonate finally forms a precipitate [59]. Cd ions are converted into a carbonate-bound form, which has stronger stability and lower toxicity than Cd ions. Calcite–CdCO3 can stably precipitate Cd. UPC also has stronger environmental adaptability [59]. Since Cd carbonate can be re-dissolved under acidic conditions, in order to maintain the stability of the carbonate precipitation formed by the Cd ions, microorganisms with stable activity and that are capable of living in the alkaline environment are required. Under different environmental conditions, the final pH value is maintained in the range of 9.0–10.0, which is an alkaline environment that is suitable for heavy metal precipitation. These bacterial consortia secrete different compounds, and the microenvironment is adjusted to maintain the alkaline environment. The steady state of the pH and growth and reproduction can still achieve the best function, and structural integrity and efficiently induce Cd carbonate precipitation [59,62]. Compared to single strains, UPC shows a great improvement, and Cd’s carbonated precipitation efficiency remains stable [59]. Other studies have shown that bacterial consortia can reduce the Cd content in the soil and can enhance the Cd tolerance of peas [60]. This research obtained promising microbial resources for the carbonated precipitation of Cd or other harmful heavy metal pollutants in the bacterial consortium [59]. Other studies have shown that the application of bacterial consortia is more effective than the application of individual strains [28,60,63,64,65,66]. The use of bacterial consortium is a sustainable and effective strategy for soil Cd precipitation that promotes the bioremediation of Cd-contaminated farmland [28,60,63,64,65,66].

### 2.10. Efficient Bio-Adsorption of Manganese (Mn) by Bacterial Consortia

The excessive release of Mn from various industrial wastewater sources into drinking water and groundwater is considered a common environmental problem [67]. Excessive Mn intake has been shown to be related to neurotoxic effects in humans [68]. At the same time, soil contaminated with heavy metals such as Mn has become a major global environmental problem, and remediating soil contaminated with heavy metals such as Mn is an urgent social, environmental, and economic problem to be solved [69,70]. Heavy metal bioremediation plays an important role in biological systems and can effectively alleviate the harm caused by heavy metals and organic pollutants [68]. Mn usually exists in the environment in the form of reduced Mn (II) [67,68]. As a microbial filter, manganese-oxidizing bacteria (MOB) adsorb biological Mn from water and play an essential role in the bioremediation of heavy metals and organic pollution [68]. It has been reported that the *Sphingobacterium* and *Bacillus* obtained from Mn-contaminated rivulet sediment were mixed and cultured to form the MOB consortium AS [68]. The MOB consortium AS showed good Mn (II) bio-adsorption performance [68]. Additionally, the MOB consortium AS can use various carbon sources to bio-adsorb Mn (II) [68]. Without Mn (II), the surface of the MOB consortium AS appeared smooth, while with Mn (II), its surface appeared rough, and it was possible for the Mn (II) in the medium to assume an insoluble form and adsorb onto the surface of bacteria [68]. There was natural Mn oxide on the surface of the MOB, and it had the potential to adsorb Mn (II). The MOB consortium AS can use various organic compounds to bio-adsorb Mn (II) and bio-adsorb Mn in the coexistence system [68]. Using the MOB consortium was more efficient in bio-adsorbing Mn (II) [68] compared to simply using MOB bacteria. Research has reported that the MOB consortium AS has great potential in remediating heavy metals and organic pollutants polluting the environment [68]. Bacterial consortium contributed to removing Mn in soil and was beneficial to bioremediation, representing a new strategy for the efficient remediation of Mn-contaminated soil [67,71,72].

The compositions and mechanisms of all-bacteria consortia discussed in this section are shown in Table 1.

## 3. The Essential Roles of Consortia Composed of Bacteria and Fungi Strains in the Degradation of Pollutants

### 3.1. Efficient Degradation of PAHs by Microbial Consortia

Due to the potentially toxic effects of hydrocarbons on animals, humans, plants, and microorganisms, the pollution caused by hydrocarbons has become an environmental problem worldwide. The continuous pollution of crude oil and its derivatives has accelerated the deposition and accumulation of foreign organisms and toxic compounds in soil [1,73,74,75]. PAHs are considered priority environmental pollutants due to their high toxicity and persistence [1,76]. PAHs are toxic, mutagenic, and teratogenic in soil. In the past century, the number of PAHs discharged into the environment by human activities has been increasing, and microbial degradation is considered the main method through which hydrocarbons degrade naturally in soil [77]. Bioremediation technology based on the use of microorganisms use to degrade pollutants is highly efficient and cost-effective. Studies have reported that microorganisms (such as bacteria, fungi, and algae) have specific catabolic activities and that they can be used to repair soil and water affected by low-molecular-weight and high-molecular-weight PAHs [78].

Studies have shown that a consortium comprising four fungal (*Aspergillus* *flavus* H6, *Aspergillus nomius* H7, *Rhizomucor variabilis* H9, and *Trichoderma asperellum* H15) and five native bacterial strains (*Klebsiella* *pneumoniae* B1, *Bacillus* *cereus* B4, *Pseudomonas* *aeruginosa* B6, *Klebsiella* *sp.* B10, *Stenotrophomonas maltophilia* B14) in the soil degrade PAHs faster compared to when a single microorganism is used and has a higher degradation value (degradation value = (beginning PAH − remaining PAH)/beginning PAH × 100) [79,80]. Moreover, the degradation of low-molecular-weight (LMW) PAHs can promote the degradation of high-molecular-weight (HMW)-PAHs and can increase the metabolic degradation of LMW PAHs and their mixtures, such as pyrene (Pyr) and benzo[a]pyrene (BaP) [78]. This is due to the increased synergistic metabolic degradation of this bacterial consortium. This bacterial consortium was inoculated to degrade the PAHs and produced noticeable microbial diversity changes in soil that had been contaminated by PAHs, which caused the microbial community to shift in the direction of aromatic hydrocarbons and intermediate degradation pathways, which greatly facilitated PAH mineralization and the removal of PAHs [78]. The mixed microbial community was able to effectively degrade a large number of PAHs in the soil, which could have been due to increased co-metabolic degradation [78]. Other studies have shown that for individual bacterial strains that can only metabolize PAHs in a limited range, heterogeneous populations with a high enzyme capacity are needed to accelerate and expand the biodegradation of PAHs. Compared to the use of a single bacterial culture, a bacterial consortium composed of four different strains of *P. aeruginosa* (PA-OBP1, PA-OBP2, PA-OBP3, and PA-OBP4) can secrete a wider range of enzymes to catalyze a degradation process that involves various reactions. The main mechanism involved the co-metabolic behavior and synergistic interaction between bacterial consortium members, promoting the degradation of PAHs. The metabolic intermediate produced by a bacterial strain can be used by other members of the bacterial consortium as a substrate for its growth and biosurfactant production [81]. Other studies have also shown that BioTiger, a patented microbial consortium of twelve natural environmental isolates, can adhere to PAHs through co-metabolism such as through the produced biosurfactant and can enhance the degradation of PAHs. This natural microbial consortium has good potential for the in situ bioremediation of tailings [82].

### 3.2. Enhanced Removal of Bisphenol S (BPS) by Microbial Consortia

In recent years, the BPS production and emissions have increased substantially [83,84,85]. BPS has shown increasing cytotoxicity in humans, including immunotoxicity, reproductive and developmental toxicity, and neurotoxicity [83,84]. BPS can significantly inhibit the immune regulation of genes [84]. Studies have shown that BPS is also common in the environment [83]. BPS has a toxic effect on soil biochemical activity [84]. Degrading BPS can eliminate ecological system pollution and can restore the balance of the global soil environment [84].

According to the European Patent Office and the global International Patent Classification (IPC) databases, the substances that are the most effective in the bioremediation of organic pollutants mainly include bacteria (57%), enzymes (19%), fungi (13%), algae (6%), and plants (4%) [84]. Among them, the enzymes that are used in enzyme bioremediation are derived from various species such as bacteria, fungi, algae, and plants. They are directly dissolved in the pore water or are immobilized with 2D and 3D super-large molecular structures and are then used for the bioremediation of pollutants. The most commonly used are proteases, cellulases, lipases, laccases, and peroxidases [86], such as carbamate pesticide degrading enzymes and the 2,4-dinitroanisole hydrolase [84,86,87]. Simultaneously, the use of bacterial and fungal consortia is a novel technology that can effectively improve the degradation of pollutants [84]. Research has shown that by combining a bacterial consortium consisting of *Pseudomonas umsongensis*, *Bacillus mycoides*, *Bacillus weihenstephanensis,* and *Bacillus subtilis* and a fungal consortium consisting of *Mucor circinelloides*, *Penicillium daleae*, *Penicillium chrysogenum,* and *Aspergillus niger*, the potential adverse effects of BPS degradation can be eliminated [84]. At the same time, studies have found that BPS can significantly inhibit soil enzyme activity and soil fertility [84]. Among these enzymes, BPS inhibits dehydrogenases and acid phosphatase the most obviously. Studies have shown that the bacterial consortium counteracts the harmful effects of BPS on the soil by enhancing catalase, urease, acid phosphatase, and alkaline phosphatase activity. Additionally, the fungal consortium can enhance the dehydrogenase, arylsulfatase, *β*-glucosidase, and acid phosphatase activity [84].

Another study reported on a bacterial consortium that was enriched from river sediment and that was mainly composed of four bacterial genera, *Hyphomicrobium*, *Pandoraea*, *Rhodococcus,* and *Cupriavidus*, which were present at relative abundances of 5.1%–52.8%. This bacterial consortium was highly efficient in degrading BPS (at pH 7 and at a temperature 30 °C, 99% of BPS with an initial concentration of 50 mg/L can be removed within 10 days) [83]. The main mechanism through was this was possible was due to the bacterial consortium being resistant to BPS and being able use BPS as a substrate. When the BPS degradation rate increased, the growth rate of the bacteria in the bacterial consortium increased, which further enhanced BPS degradation [83]. In addition, the optimal pH (7) and temperature (30 °C) for the growth of the bacterial consortium using BPS as the sole substrate are feasible in the common environment, allowing this bacterial consortium to be used in multiple applications, such as in the restoration of soil contaminated by BPS [83]. Compared to this bacterial consortium, the BPS degradation rate in a single culture was lower [83]. For example, compared to the bacterial consortium, the two degradation strains *Terrimonas pekingensis* and *Pseudomonas* sp. had much lower BPS degradation efficiencies [83].

### 3.3. Enhanced Degradation of Bisphenol F (BPF) by Microbial Consortia

BPF is a dihydroxydiphenylmethane of the diphenylalkene family [88,89]. It is widely used to produce sewage pipes, adhesives, dental sealants, and acetonitrile [89]. It has become a major pollutant due to leaking from the industrial products containing it [88].

BPF is a toxic soil pollutant that inhibits the biochemical activity of soil [88]. It is necessary to effectively degrade BPF to eliminate the impact of BPF on the environment [89]. Studies have shown that a bacterial consortium composed of four bacterial genera, *Salmonella enterica* (46.4%), *Enterobacter* (28.6%), *Citrobacter* (21.4%), and *Pseudomonas* (3.6%) has good BPF removal ability [89]. Under certain conditions (35 °C, 150 rpm, C/N ratio over 10, 300 mg/L BPF), this consortium can completely degrade BPF [89]. The main mechanism for this is that this bacterial consortium can synergistically degrade BPF [89]. BPF degrades into bis(4-hydroxyphenyl)methanol and DHBP (BPF degradation intermediates), which then degrade into 4-hydroxyphenyl-4-hydroxybenzoate and 1,4-hydroquinone, which finally degrade into CO_2_ [90]. In addition, this bacterial consortium can use BPF and its metabolites as a carbon source to remove BPF, effectively biodegrading BPF [89]. The bacterial consortium mentioned above has a better BPF degradation performance than that observed for a single culture and is a potentially good method for treating sites that have been contaminated by BPF [89].

Other research reported a bacterial consortium composed of four kinds of bacteria (*Pseudomonas umsongensis*, *Bacillus mycoides*, *Bacillus weihenstephanensis,* and *Bacillus subtilis*) and a fungal consortium consisting of four fungi (*Mucor circinelloides*, *Penicillium daleae*, *Penicillium chrysongogenum*, and *Aspergillus niger*), which can efficiently degrade BPF and eliminate the damage that BPF can cause to the environment [88]. The main mechanism of this is the bacterial consortium increases the activity of urease, β-glucosidase, catalase, and alkaline phosphatase. In addition, this fungal consortium increases the activity of dehydrogenases, catalase, β-glucosidase, alkaline phosphatase, and urease. Thus, the bacterial and fungal consortia reduced the inhibitory effect of BPF on enzyme activity [88]. Compared to the fungal consortium, the bacterial consortium had a better effect on BPF-contaminated soil remediation [88]. Compared to a single culture, the bacterial and fungal consortia had higher stability and stronger metabolic potential, indicating that they had more advantages in repairing soil contaminated with BPF [88,91]]. Therefore, these bacterial and fungal consortia can efficiently biodegrade BPF, reducing the toxic effect of BPF on the biochemical activity in soil. This is a potentially effective bioremediation method for soil contaminated by BSF [88], eliminating BPF pollution in the ecosystem and restoring the balance of the global soil environment [84,88].

### 3.4. Effecient Degradation of Crude Oil by Microbial Consortia

Due to the continuous growth of energy demands and the innovation of oil recovery technology, the extraction, refining, and use of crude oil worldwide is proliferating [92]. Due to the complex composition of crude oil, its poor fluidity, and its biological toxicity, environmental petroleum pollution has become a continuous threat to human society and to the natural environment [73,75,77]. Due to its low cost, environmental friendliness, and ability to fully degrade pollutants, bioremediation is considered to be one of the most promising methods to treat crude oil contamination [8,93].

The co-cultivation of indigenous microorganisms and exogenous microorganisms is an effective biological method to improve the metabolic ability of microorganisms and the synergistic degradation ability of crude oil [18]. *Scedosporium boydii* has been used to degrade various petroleum pollutants and has been proven to be an exogenous strain that can enhance the degradation ability of environmental microorganisms [93]. This study showed that the co-culture of a microbial consortium composed of indigenous bacteria, the main members of which were *Paraburkholderia* sp. and *Paraburkholderia tropica* and the exogenous fungus *Scedosporium boydii*, was able to significantly enhance the biodegradation of crude oil [93]. Co-cultivation can simultaneously increase the degradation rate of *n*-alkanes, aromatic fractions, and crude oil [93]. In particular, the inoculation ratio of bacteria to fungi was 3:1, and the degradation rate of crude oil increased from 61.06% to 81.45% under certain co-cultivation conditions [93]. After inoculating the microbial consortium composed of indigenous bacteria and an exogenous fungus, *Scedosporium boydii*, the microbial activity was significantly enhanced, and the uniformity and diversity of bacteria in the prescribed co-culture increased [93]. The co-degradation of crude oil in the bacterial and fungal consortium was beneficial to the bioremediation of petroleum-contaminated soil [93]. The study showed that the microbial consortium degraded crude oil well and that it had good potential for applications related to the remediation of crude oil-contaminated environments [94,95,96,97,98,99,100,101]. The composition and mechanisms of the bacterial and fungal consortia are shown in Table 2.

## 4. Enhancement of Degradation Efficiency by Adding Chemicals to Microbial Consortia

Soil pollution caused by crude oil is a severe environmental problem that is mainly caused by accidental spillage and the discharge of petroleum or oily waste and poses a potential risk to human health [102]. Therefore, many studies have been conducted to explore practical techniques for removing crude oil from contaminated soil [98]. Among these technologies, bioremediation has broad development prospects due to its non-invasive and cost-effective characteristics [97].

Studies have shown that the addition of two surfactants, nonionic surfactant polyoxyethylene sorbitan monooleate (Tween 80) and anionic surfactant sodium dodecyl sulfate (SDS), can enhance the biodegradation of crude oil by means of a mixed bacterial consortium in the soil–water system [97,103,104,105]. A mixed bacterial consortium was obtained from the activated sludge of a cooking plant and was composed of *Alphaproteobacteria* (42%) and *Gammaproteobacteria* (35%), *Mycobacterium* sp. (12%), and unclassified *rhizobiales* (11%), of which *Rhodanobacter* sp. was the dominant species, accounting for 34% [97]. Both Tween 80 and SDS can be used as carbon sources and can promote mixed bacterial consortium growth [97]. Crude oil degradation can be enhanced by adding Tween 80 and SDS [97]. The crude oil degradation performance of Tween 80 was generally better than that of SDS [97]. Studies have shown that adding Tween 80 and inoculating it with a mixed bacterial consortium has a better purification effect on crude oil-contaminated soil [97]. Simultaneously, the biodegradation effect of the mixed bacterial consortium was better than that of pure bacteria [97]. Additionally, studies have also shown that the degradation efficiency of the crude oil treated with the Tween 20 surfactant was higher than that of the bacterial consortium not treated with Tween 20 [106]. The addition of biosurfactants to crude oil-contaminated soils, on the one hand, improves the desorption and subsequent dissolution of pollutants from solid substrates to aqueous solutions. On the other hand, biosurfactants also act as carbon sources, helping to stimulate cell growth and the microbial activity of a bacterial consortium, accelerating the biodegradation process through co-metabolism [107]. The combination of mixed bacterial consortium and surfactants can significantly improve the degradation efficiency of crude oil, providing a more practical choice for environmental engineers in crude oil-contaminated soil remediation [97]. Other studies also have also shown that the addition of surfactants in the bacterial consortium can enhance biodegradation and can effectively reduce the crude oil content on the sea surface, which can help to develop the bioremediation strategy of crude oil in the marine ecosystem [106,108,109,110]. This is a new method of using the bacterial consortium for ecological restoration [97,106,108,109,110]. Studies have also shown that *Sapindus* saponins (natural surfactants) can modify bacterial cell properties and reduce cells hydrophobicity, and changing the electrokinetic behavior of cells may thus be advantageous to support the treatment of recently crude oil-contaminated soils [111].

Other amendment methods have also been developed to enhance bacterial consortium activity. It has been reported that the addition of biochar to bacterial consortia can stimulate microbial activity and can enhance the bacterial consortium’s ability to degrade pollutants [112]. The inoculation of biochar may change the soil structure and increase the biodegradation rate of organic matter [112]. In addition, adding immobilized laccase to the bacterial consortium can also effectively enhance the bacterial consortium’s bioremediation ability, which is due to the fact that immobilized laccase is beneficial to the growth and metabolism of the bacterial consortium [113]. Additionally, adding washing agents consisting of a lipopeptide biosurfactant (in foamate or cell-free broth), Dehydol LS7TH (fatty alcohol ethoxylate 7EO, oleochemical surfactant), butanol (as a lipophilic linker), and biochar to the bacterial consortium can enhance the bioactivity of the bacterial consortium, which can also promote crude oil degradation [114]. Moreover, the degradation of pollutants can also be enhanced using natural raw materials [115]. Furthermore, the application of digestate and fly ash can greatly enhance microbial activity and diversity, which can be successfully used to remediate contaminated soils [116]. Therefore, by adding biochar or immobilized laccase or washing agents, as well as digestate and fly ash to the bacterial consortium, the biodegradation of soil pollutants via the bacterial consortium could be significantly improved.

## 5. Discussion and Conclusions

Although the amounts of these pollutants in the soil varies from place to place, the increase in synthetic, chemical, industrial, agricultural, and residential uses has led to the release of large amounts of organic and inorganic pollutants into the soil. Among them, the most important organic pollutants are PAHs, DBP, and DOP in petroleum, and the most important inorganic pollutants are heavy metals such as Cd and Mn. Therefore, reducing the soil pollution caused by heavy metals and organic pollutants and promoting the use of sustainable methods to repair these contaminated soils are very important measures to prevent environmental and health problems. Compared to other methods, biodegradation is an effective, eco-friendly method with many advantages.

Many studies have shown that it is difficult for a single strain to completely degrade pollutants and that microbial consortia have different removal abilities. A microbial consortium can integrate the advantages of each strain to achieve the effective degradation of pollutants. Compared to a single culture of microorganisms, mixed microbial consortia exhibit good substrate tolerance, and microbial consortia have better pollutant adsorption or degradation performance and are suitable for use as a promising soil pollutant bioremediation technology. The main mechanism of this technology can be summarized as the key intermediate compounds that synergistically degrade pollutants; produce large amounts of high-efficiency biosurfactants; are able to self-regulate and adapt; promote bacterial growth; produce key degrading enzymes; and enhance enhancement bacterial activity. Among them, the most common mechanism is the synergistic degradation of the key intermediate compounds of pollutants, reducing the accumulation of intermediate products, thereby increasing the metabolic pathways for the biodegradation of organic pollutants. Moreover, direct interspecific electron transfer (DIET) between homogeneous nutritional partners can be established by directly adding surfactants to promote the growth of a microbial consortium; adding biochar to stimulate microbial activity; and adding immobilized laccase to enhance the microbial growth, etc., to significantly improve the degradation efficiency of soil pollutants. By reviewing and summarizing the ability of microbial consortia to show high bio-adsorption or biodegradation or the conversion of soil pollutants into carbonate precipitation, research can provide inspiration for the construction of suitable microbial consortia for the treatment of specific soil pollutants in the future.

Bacterial consortia are effective in improving the ability of microorganisms to degrade and synthesize organic and inorganic pollutants. Further research on the metabolic pathways of pollutants in consortia will increase our scientific understanding of effective methods for removing pollutants. Microbial metabolism reduces the content of these pollutants and creates a sustainable way to reduce soil pollution. It is necessary to conduct more high-throughput research on microbial technology to develop management strategies for the bioremediation of contaminated soil. The addition of a bacterial consortium to the soil will have a good impact on environmental sustainability, and it will be of great importance for the restoration of contaminated land in an environmentally friendly way and will open up a new way for sustainable development.

## Figures and Tables

**Table 1:** Composition and mechanism of all-bacteria consortia.

Contaminant	The Microorganisms Involved	Detailed Mechanism of Synergy	Mechanism Type	References
Pyrene	Five cultivable bacteria (*Mycobacterium* spp. PO1 and PO2, *Novosphingobium pentaromativorans* PY1, *Ochrobactrum* sp. PW1, and *Bacillus* sp. FW1)	Degrade the essential intermediate compounds produced during the degradation of pyrene by other members. Degrade phthalates or protocatechuates. Produce a large amount of high-efficiency biosurfactant, thereby increasing the soluble pyrene content, improving its bioavailability and biodegradability.	1,2	[21]
DBP	*Pandoraea* sp. and *Microbacterium* sp.	Degrade dimethyl phthalate (DMP), di-2-ethylhexyl phthalate (DEHP), and phthalic acid (PA); one strain of bacteria uses the intermediates produced by another strain.	1	[32]
DOP	*Arthrobacter* sp. SLG-4 and *Rhodococcus* sp. SLG-6	Metabolize DOP as phthalic acid (PA). Degrade DOP to PA and then metabolize it to protocatechuate acid and finally to tricarboxylic acid (TCA) through the meta-cleavage pathway.	1	[35]
TPHP	*Pseudarthrobacter*, *Sphingopyxis*, *Methylobacterium,* and *Pseudomonas*	Synergy between the different strains of the microbial consortium GYY. Self-regulate and adapt during the degradation of TPHP.	1,3	[36]
PHs	*Diaphorobacter* sp. strain LR2014-1 and *Achromobacter* sp. strain ANB-1	Synergistic catabolism of linuron leads to a more effective catabolism of linuron and promotes growth. It contains two evolutionarily different hydrolases, the amide hydrolase superfamily Phh, and the amidase superfamily TccA2, which have complementary roles in the hydrolysis of different types of PHs.	1,5	[43]
Butane	Six butane-oxidizing bacteria (BOB, PG-3-1, PG-3-6, PG-3-2, PG-3-10, PG-3-7, and PG-3-12) and butanol oxidizing strain *Mycobacterium* sp. PG-3-5	The BOB strain oxidized butane to butanol. The butanol oxidizing strain removed butanol, promoting the growth of both BOB and butanol-oxidizing bacteria. The co-cultivation of BOB strains and butanol-oxidizing strains has a synergistic effect.	1,4	[47]
DON	*Pseudomonas* sp. Y1 and *Lysobacter* sp. S1	Convert DON into non-toxic 3-epi-DON. DON degradation was the DON epimerization process. DON epimerization is a two-step enzymatic detoxification pathway of DON. The synergistic metabolism promoted DON epimerization.	1	[51]
TC	*Raoultella* sp. XY-1 and *Pandoraea* sp. XY-2	The presence of a biochemical synergistic effect between the bacteria, which enhanced the bacteria’s activity and the degradation of TC.	6	[56]
Cd	UPC (70.22–75.41% of *Sporosarcina*, 13.83–20.66% of norank_f_*Bacillaceae*, and 5.91–13.69% of unclassified_f_*Bacillaceae*)	Contained a bacterial consortium with high urease activity and carbonate formation ability. In the urease hydrolysis process of urea, Cd^2+^ cations are adsorbed through their negative charges, and the combination of Cd^2+^ and carbonate finally formed a precipitate. Stronger environmental adaptability.	3	[59]
Mn	*Sphingobacterium* and *Bacillus*	Use various organic compounds to bio-adsorb Mn (II) and bio-adsorb Mn in the coexistence system.	3	[68]

**Table 2:** Composition and mechanisms of bacterial and fungal consortia.

Contaminant	Microorganisms Involved	Detailed Mechanism of Synergy	Mechanism Type	References
PAHs	Four fungal (*Aspergillus flavus* H6, *Aspergillus nomius* H7, *Rhizomucor variabilis* H9, *Trichoderma asperellum* H15) and five native bacterial strains (*Klebsiella pneumoniae* B1, *Bacillus cereus* B4, *Pseudomonas aeruginosa* B6, *Klebsiella* sp. B10, *Stenotrophomonas maltophilia* B14)	Increase synergistic metabolic degradation. Produced noticeable microbial diversity changes in the soil contaminated by PAHs, which caused the microbial community to shift in the direction of aromatic hydrocarbons and intermediate degradation pathways.	1	[78]
	Four different strains of *P. aeruginosa* (PA-OBP1, PA-OBP2, PA-OBP3, and PA-OBP4)	Secrete a wider range of enzymes to catalyze the degradation process through various reactions.	5	[81]
BPS	A bacterial consortium consisting of *Pseudomonas umsongensis*, *Bacillus mycoides*, *Bacillus weihenstephanensis,* and *Bacillus subtilis* and a fungal consortium consisting of *Mucor circinelloides*, *Penicillium daleae*, *Penicillium chrysogenum,* and *Aspergillus niger*	Counteract the harmful effects of BPS on the soil by enhancing the activity of catalase, urease, acid phosphatase, and alkaline phosphatase. Enhance the activity of dehydrogenase, arylsulfatase, β-glucosidase, and acid phosphatase.	5	[84]
	Four bacterial genera *Hyphomicrobium*, *Pandoraea*, *Rhodococcus,* and *Cupriavidus*	Resistant to BPS and can use BPS as a substrate. When the degradation rate of BPS increased, the growth rate of the bacteria increased.	4	[83]
BPF	Four bacterial genera, *Salmonella enterica* (46.4%), *Enterobacter* (28.6%), *Citrobacter* (21.4%), and *Pseudomonas* (3.6%)	Synergistically degrade BPF. Use BPF and its metabolites as a carbon source to remove BPF.	1,4	[89]
	Four kinds of bacteria (*Pseudomonas umsongensis*, *Bacillus mycoides*, *Bacillus weihenstephanensis,* and *Bacillus subtilis*) and four fungi (*Mucor circinelloides*, *Penicillium daleae*, *Penicillium chrysongogenum,* and *Aspergillus niger*)	Increased the activity of urease, β-glucosidase, catalase, and alkaline phosphatase. Increased the activity of dehydrogenases, catalase, β-glucosidase, alkaline phosphatase, and urease.	5	[88]
Crude oil	Indigenous bacterial *Paraburkholderia* sp. and *Paraburkholderia tropica* and exogenous fungus *Scedosporium boydii*	Significantly enhanced microbial activity and increased the uniformity and diversity.	6	[93]

## References

[1] Ma M., Zheng L., Yin X., Gao W., Han B., Li Q., Zhu A., Chen H., Yang H. (2021). Reconstruction and evaluation of oil-degrading consortia isolated from sediments of hydrothermal vents in the South Mid-Atlantic Ridge. Sci. Rep..

[2] Bhatt P., Gangola S., Bhandari G., Zhang W., Maithani D., Mishra S., Chen S. (2021). New insights into the degradation of synthetic pollutants in contaminated environments. Chemosphere.

[3] Abidi M.A., Hairom N.H.H., Madon R.H., Kassim A.S.M., Sidik D.A.B., Al-Gheethi A.A.S. (2020). Optimization Of Microbial Consortium (AB-101) Performance In Palm Oil Mill Effluent (POME) Treatment Via Response Surface Methodology (RSM). Biointerface Res. Appl. Chem..

[4] Tian F., Wang Y., Guo G., Ding K., Yang F., Wang H., Cao Y., Liu C. (2021). Enhanced azo dye biodegradation at high salinity by a halophilic bacterial consortium. Bioresour. Technol..

[5] Jeyasundar P., Ali A., Azeem M., Li Y., Guo D., Sikdar A., Abdelrahman H., Kwon E., Antoniadis V., Mani V.M. (2021). Green remediation of toxic metals contaminated mining soil using bacterial consortium and *Brassica juncea*. Environ. Pollut..

[6] Avila R., Peris A., Eljarrat E., Vicent T., Blanquez P. (2021). Biodegradation of hydrophobic pesticides by microalgae: Transformation products and impact on algae biochemical methane potential. Sci. Total Environ..

[7] Shakeri F., Babavalian H., Amoozegar M.A., Ahmadzadeh Z., Zuhuriyanizadi S., Afsharian M.P. (2020). Production and Application of Biosurfactants in Biotechnology. Biointerface Res. Appl. Chem..

[8] Varjani S., Pandey A., Upasani V.N. (2021). Petroleum sludge polluted soil remediation: Integrated approach involving novel bacterial consortium and nutrient application. Sci. Total Environ..

[9] Kang D., Huang Y., Nesme J., Herschend J., Jacquiod S., Kot W., Hansen L.H., Lange L., Sorensen S.J. (2021). Metagenomic analysis of a keratin-degrading bacterial consortium provides insight into the keratinolytic mechanisms. Sci. Total Environ..

[10] Ali S.S., Mustafa A.M., Kornaros M., Sun J., Khalil M., El-Shetehy M. (2021). Biodegradation of creosote-treated wood by two novel constructed microbial consortia for the enhancement of methane production. Bioresour. Technol..

[11] McGachy L., Skarohlid R., Martinec M., Roskova Z., Smrhova T., Strejcek M., Uhlik O., Marek J. (2021). Effect of chelated iron activated peroxydisulfate oxidation on perchloroethene-degrading microbial consortium. Chemosphere.

[12] Zielinski M., Zielinska M., Cydzik-Kwiatkowska A., Rusanowska P., Debowski M. (2021). Effect of static magnetic field on microbial community during anaerobic digestion. Bioresour. Technol..

[13] Ali S.S., Kornaros M., Manni A., Sun J., El-Shanshoury A.E.R., Kenawy E.R., Khalil M.A. (2020). Enhanced anaerobic digestion performance by two artificially constructed microbial consortia capable of woody biomass degradation and chlorophenols detoxification. J. Hazard. Mater..

[14] Vieira G.A.L., Cabral L., Otero I.V.R., Ferro M., Faria A.U., Oliveira V.M., Bacci M., Sette L.D. (2021). Marine associated microbial consortium applied to RBBR textile dye detoxification and decolorization: Combined approach and metatranscriptomic analysis. Chemosphere.

[15] Basak B., Patil S.M., Saha S., Kurade M.B., Ha G.S., Govindwar S.P., Lee S.S., Chang S.W., Chung W.J., Jeon B.H. (2021). Rapid recovery of methane yield in organic overloaded-failed anaerobic digesters through bioaugmentation with acclimatized microbial consortium. Sci. Total Environ..

[16] Liu Z., Zhou A., Wang S., Cheng S., Yin X., Yue X. (2021). Quorum sensing shaped microbial consortia and enhanced hydrogen recovery from waste activated sludge electro-fermentation on basis of free nitrous acid treatment. Sci. Total Environ..

[17] Zhang C., Wu X., Wu Y., Li J., An H., Zhang T. (2021). Enhancement of dicarboximide fungicide degradation by two bacterial co-cultures of *Providencia stuartii* JD and *Brevundimonas naejangsanensis* J3. J. Hazard. Mater..

[18] Liu J., Zhao B., Lan Y., Ma T. (2021). Enhanced degradation of different crude oils by defined engineered consortia of *Acinetobacter venetianus* RAG-1 mutants based on their alkane metabolism. Bioresour. Technol..

[19] Abou Khalil C., Prince V.L., Prince R.C., Greer C.W., Lee K., Zhang B., Boufadel M.C. (2021). Occurrence and biodegradation of hydrocarbons at high salinities. Sci. Total Environ..

[20] Lipińska A., Wyszkowska J., Kucharski J. (2021). Microbiological and Biochemical Activity in Soil Contaminated with Pyrene Subjected to Bioaugmentation. Water Air Soil Pollut..

[21] Wanapaisan P., Laothamteep N., Vejarano F., Chakraborty J., Shintani M., Muangchinda C., Morita T., Suzuki-Minakuchi C., Inoue K., Nojiri H. (2018). Synergistic degradation of pyrene by five culturable bacteria in a mangrove sediment-derived bacterial consortium. J. Hazard. Mater..

[22] Sarma H., Sonowal S., Prasad M.N.V. (2019). Plant-microbiome assisted and biochar-amended remediation of heavy metals and polyaromatic compounds horizontal line a microcosmic study. Ecotoxicol. Environ. Saf..

[23] Zhang S., Hu Z., Wang H. (2019). Metagenomic analysis exhibited the co-metabolism of polycyclic aromatic hydrocarbons by bacterial community from estuarine sediment. Environ. Int..

[24] Wang C., Gu L., Ge S., Liu X., Zhang X., Chen X. (2019). Remediation potential of immobilized bacterial consortium with biochar as carrier in pyrene-Cr(VI) co-contaminated soil. Environ. Technol..

[25] Bellino A., Baldantoni D., Picariello E., Morelli R., Alfani A., De Nicola F. (2019). Role of different microorganisms in remediating PAH-contaminated soils treated with compost or fungi. J. Environ. Manag..

[26] Qiao K., Tian W., Bai J., Wang L., Zhao J., Song T., Chu M. (2020). Removal of high-molecular-weight polycyclic aromatic hydrocarbons by a microbial consortium immobilized in magnetic floating biochar gel beads. Mar. Pollut. Bull..

[27] Madrid F., Rubio-Bellido M., Villaverde J., Pena A., Morillo E. (2019). Natural and assisted dissipation of polycyclic aromatic hydrocarbons in a long-term co-contaminated soil with creosote and potentially toxic elements. Sci. Total Environ..

[28] Kotoky R., Pandey P. (2020). Difference in the rhizosphere microbiome of Melia azedarach during removal of benzo(a)pyrene from cadmium co-contaminated soil. Chemosphere.

[29] Vaidya S., Devpura N., Jain K., Madamwar D. (2018). Degradation of Chrysene by Enriched Bacterial Consortium. Front. Microbiol..

[30] Bai N., Li S., Zhang J., Zhang H., Zhang H., Zheng X., Lv W. (2020). Efficient biodegradation of DEHP by CM9 consortium and shifts in the bacterial community structure during bioremediation of contaminated soil. Environ. Pollut..

[31] Wang Y., Li F., Ruan X., Song J., Lv L., Chai L., Yang Z., Luo L. (2017). Biodegradation of di-n-butyl phthalate by bacterial consortium LV-1 enriched from river sludge. PLoS ONE.

[32] Yang J., Guo C., Liu S., Liu W., Wang H., Dang Z., Lu G. (2018). Characterization of a di-n-butyl phthalate-degrading bacterial consortium and its application in contaminated soil. Environ. Sci. Pollut. Res. Int..

[33] Li F., Liu Y., Wang D., Zhang C., Yang Z., Lu S., Wang Y. (2018). Biodegradation of di-(2-ethylhexyl) phthalate by a halotolerant consortium LF. PLoS ONE.

[34] Wang Y., Zhan W., Liu Y., Cheng S., Zhang C., Ma J., Chen R. (2021). Di-n-octyl phthalate degradation by a halotolerant bacterial consortium LF and its application in soil. Environ. Technol..

[35] Zhang K., Liu Y., Chen Q., Luo H., Zhu Z., Chen W., Chen J., Mo Y. (2018). Biochemical pathways and enhanced degradation of di-n-octyl phthalate (DOP) in sequencing batch reactor (SBR) by *Arthrobacter* sp. SLG-4 and *Rhodococcus* sp. SLG-6 isolated from activated sludge. Biodegradation.

[36] Yang Y., Yin H., Peng H., Lu G., Dang Z. (2020). Biodegradation of triphenyl phosphate using an efficient bacterial consortium GYY: Degradation characteristics, metabolic pathway and 16S rRNA genes analysis. Sci. Total Environ..

[37] Wang J., Khokhar I., Ren C., Li X., Wang J., Fan S., Jia Y., Yan Y. (2019). Characterization and 16S metagenomic analysis of organophosphorus flame retardants degrading consortia. J. Hazard. Mater..

[38] Wang J., Li X., Wu W., Fan S., Jia Y., Wang J., Yan Y. (2019). Characterization and 16S rRNA gene-based metagenomic analysis of the organophosphorous flame retardants degrading consortium YC-BJ1. Sheng Wu Gong Cheng Xue Bao.

[39] Wang J., Hlaing T.S., Nwe M.T., Aung M.M., Ren C., Wu W., Yan Y. (2021). Primary biodegradation and mineralization of aryl organophosphate flame retardants by *Rhodococcus-Sphingopyxis* consortium. J. Hazard. Mater..

[40] Villaverde J., Rubio-Bellido M., Merchan F., Morillo E. (2017). Bioremediation of diuron contaminated soils by a novel degrading microbial consortium. J. Environ. Manag..

[41] Zhang L., Hu Q., Liu B., Li F., Jiang J.D. (2020). Characterization of a Linuron-Specific Amidohydrolase from the Newly Isolated Bacterium *Sphingobium* sp. Strain SMB. J. Agric. Food Chem..

[42] Ozturk B., Werner J., Meier-Kolthoff J.P., Bunk B., Sproer C., Springael D. (2020). Comparative Genomics Suggests Mechanisms of Genetic Adaptation toward the Catabolism of the Phenylurea Herbicide Linuron in *Variovorax*. Genome Biol. Evol..

[43] Zhang L., Hang P., Hu Q., Chen X.L., Zhou X.Y., Chen K., Jiang J.D. (2018). Degradation of Phenylurea Herbicides by a Novel Bacterial Consortium Containing Synergistically Catabolic Species and Functionally Complementary Hydrolases. J. Agric. Food Chem..

[44] Sorensen S.R., Albers C.N., Aamand J. (2008). Rapid mineralization of the phenylurea herbicide diuron by *Variovorax* sp. strain SRS16 in pure culture and within a two-member consortium. Appl. Environ. Microbiol..

[45] Villaverde J., Posada-Baquero R., Rubio-Bellido M., Laiz L., Saiz-Jimenez C., Sanchez-Trujillo M.A., Morillo E. (2012). Enhanced mineralization of diuron using a cyclodextrin-based bioremediation technology. J. Agric. Food Chem..

[46] Yin X., Luan X., Xu A., Li Q., Cui Z., Valentine D.L. (2018). Genome Sequence of a Marine Alkane Degrader, *Alcanivorax* sp. Strain 97CO-6. Genome Announc..

[47] Zhang Y., Deng C.P., Shen B., Yang J.S., Wang E.T., Yuan H.L. (2016). Syntrophic Interactions Within a Butane-Oxidizing Bacterial Consortium Isolated from Puguang Gas Field in China. Microb. Ecol..

[48] Holler T., Widdel F., Knittel K., Amann R., Kellermann M.Y., Hinrichs K.U., Teske A., Boetius A., Wegener G. (2011). Thermophilic anaerobic oxidation of methane by marine microbial consortia. ISME J..

[49] Rajwar D., Paliwal R., Rai J.P.N. (2017). Biodegradation of pulp and paper mill effluent by co-culturing ascomycetous fungi in repeated batch process. Environ. Monit. Assess..

[50] Wang Y., Wang G., Dai Y., Wang Y., Lee Y.W., Shi J., Xu J. (2019). Biodegradation of Deoxynivalenol by a Novel Microbial Consortium. Front. Microbiol..

[51] Zhai Y., Zhong L., Gao H., Lu Z., Bie X., Zhao H., Zhang C., Lu F. (2019). Detoxification of Deoxynivalenol by a Mixed Culture of Soil Bacteria With 3-epi-Deoxynivalenol as the Main Intermediate. Front. Microbiol..

[52] Wang G., Wang Y., Man H., Lee Y.W., Shi J., Xu J. (2020). Metabolomics-guided analysis reveals a two-step epimerization of deoxynivalenol catalyzed by the bacterial consortium IFSN-C1. Appl. Microbiol. Biotechnol..

[53] Tan J., De Zutter N., De Saeger S., De Boevre M., Tran T.M., van der Lee T., Waalwijk C., Willems A., Vandamme P., Ameye M. (2021). Presence of the Weakly Pathogenic *Fusarium poae* in the *Fusarium* Head Blight Disease Complex Hampers Biocontrol and Chemical Control of the Virulent Fusarium graminearum Pathogen. Front. Plant. Sci..

[54] He W.J., Shi M.M., Yang P., Huang T., Yuan Q.S., Yi S.Y., Wu A.B., Li H.P., Gao C.B., Zhang J.B. (2020). Novel Soil Bacterium Strain *Desulfitobacterium* sp. PGC-3-9 Detoxifies Trichothecene Mycotoxins in Wheat via De-Epoxidation under Aerobic and Anaerobic Conditions. Toxins (Basel).

[55] Liu C.X., Xu Q.M., Yu S.C., Cheng J.S., Yuan Y.J. (2020). Bio-removal of tetracycline antibiotics under the consortium with probiotics *Bacillus clausii* T and *Bacillus amyloliquefaciens* producing biosurfactants. Sci Total Environ..

[56] Wu X., Gu Y., Wu X., Zhou X., Zhou H., Amanze C., Shen L., Zeng W. (2020). Construction of a Tetracycline Degrading Bacterial Consortium and Its Application Evaluation in Laboratory-Scale Soil Remediation. Microorganisms.

[57] Kumar A., Tripti, Maleva M., Bruno L.B., Rajkumar M. (2021). Synergistic effect of ACC deaminase producing *Pseudomonas* sp. TR15a and siderophore producing *Bacillus aerophilus* TR15c for enhanced growth and copper accumulation in *Helianthus annuus* L.. Chemosphere.

[58] Wang Q., Li X., Yang Q., Chen Y., Du B. (2019). Evolution of microbial community and drug resistance during enrichment of tetracycline-degrading bacteria. Ecotoxicol. Environ. Saf..

[59] Yin T., Lin H., Dong Y., Li B., He Y., Liu C., Chen X. (2021). A novel constructed carbonate-mineralized functional bacterial consortium for high-efficiency cadmium biomineralization. J. Hazard. Mater..

[60] Belimov A.A., Shaposhnikov A.I., Azarova T.S., Makarova N.M., Safronova V.I., Litvinskiy V.A., Nosikov V.V., Zavalin A.A., Tikhonovich I.A. (2020). Microbial Consortium of PGPR, Rhizobia and Arbuscular Mycorrhizal Fungus Makes Pea Mutant SGECd(t) Comparable with Indian Mustard in Cadmium Tolerance and Accumulation. Plants (Basel).

[61] Talukdar D., Jasrotia T., Sharma R., Jaglan S., Kumar R., Vats R., Kumar R., Mahnashi M.H., Umar A. (2020). Evaluation of novel indigenous fungal consortium for enhanced bioremediation of heavy metals from contaminated sites. Environ. Technol. Innov..

[62] Padan E., Bibi E., Ito M., Krulwich T.A. (2005). Alkaline pH homeostasis in bacteria: New insights. Biochim. Biophys. Acta.

[63] Liao Q., He L., Tu G., Yang Z., Yang W., Tang J., Cao W., Wang H. (2021). Simultaneous immobilization of Pb, Cd and As in soil by hybrid iron-, sulfate- and phosphate-based bio-nanocomposite: Effectiveness, long-term stability and bioavailablity/bioaccessibility evaluation. Chemosphere.

[64] Tang L., Hamid Y., Zehra A., Sahito Z.A., He Z., Beri W.T., Khan M.B., Yang X. (2020). Fava bean intercropping with Sedum alfredii inoculated with endophytes enhances phytoremediation of cadmium and lead co-contaminated field. Environ. Pollut..

[65] Tang L., Hamid Y., Zehra A., Shohag M.J.I., He Z., Yang X. (2020). Endophytic inoculation coupled with soil amendment and foliar inhibitor ensure phytoremediation and argo-production in cadmium contaminated soil under oilseed rape-rice rotation system. Sci. Total Environ..

[66] Ahsan M.T., Tahseen R., Ashraf A., Mahmood A., Najam-Ul-Haq M., Arslan M., Afzal M. (2019). Effective plant-endophyte interplay can improve the cadmium hyperaccumulation in *Brachiaria mutica*. World J. Microbiol. Biotechnol..

[67] Hou D., Zhang P., Wei D., Zhang J., Yan B., Cao L., Zhou Y., Luo L. (2020). Simultaneous removal of iron and manganese from acid mine drainage by acclimated bacteria. J. Hazard. Mater..

[68] Wan W., Xing Y., Qin X., Li X., Liu S., Luo X., Huang Q., Chen W. (2020). A manganese-oxidizing bacterial consortium and its biogenic Mn oxides for dye decolorization and heavy metal adsorption. Chemosphere.

[69] Luo Z., Tian D., Ning C., Yan W., Xiang W., Peng C. (2015). Roles of *Koelreuteria bipinnata* as a suitable accumulator tree species in remediating Mn, Zn, Pb, and Cd pollution on Mn mining wastelands in southern China. Environ. Earth Sci..

[70] Baltrenaite E., Baltrenas P., Lietuvninkas A., Sereviciene V., Zuokaite E. (2014). Integrated evaluation of aerogenic pollution by air-transported heavy metals (Pb, Cd, Ni, Zn, Mn and Cu) in the analysis of the main deposit media. Environ. Sci. Pollut. Res. Int..

[71] Moradtalab N., Ahmed A., Geistlinger J., Walker F., Hoglinger B., Ludewig U., Neumann G. (2020). Synergisms of Microbial Consortia, N Forms, and Micronutrients Alleviate Oxidative Damage and Stimulate Hormonal Cold Stress Adaptations in Maize. Front. Plant. Sci..

[72] Nadhirawaty R., Titah H.S. (2019). Simultaneous Bioaugmentation and Biostimulation to Remediate Soil Contaminated by Ship Dismantling in Bangkalan District, Indonesia. J. Health Pollut..

[73] Mori J.F., Kanaly R.A. (2020). Multispecies Diesel Fuel Biodegradation and Niche Formation Are Ignited by Pioneer Hydrocarbon-Utilizing Proteobacteria in a Soil Bacterial Consortium. Appl. Environ. Microbiol..

[74] Liang J., Gao S., Wu Z., Rijnaarts H.H.M., Grotenhuis T. (2021). DNA-SIP identification of phenanthrene-degrading bacteria undergoing bioaugmentation and natural attenuation in petroleum-contaminated soil. Chemosphere.

[75] Phulpoto I.A., Hu B., Wang Y., Ndayisenga F., Li J., Yu Z. (2021). Effect of natural microbiome and culturable biosurfactants-producing bacterial consortia of freshwater lake on petroleum-hydrocarbon degradation. Sci. Total Environ..

[76] Laothamteep N., Kawano H., Vejarano F., Suzuki-Minakuchi C., Shintani M., Nojiri H., Pinyakong O. (2021). Effects of environmental factors and coexisting substrates on PAH degradation and transcriptomic responses of the defined bacterial consortium OPK. Environ. Pollut..

[77] Dai X., Lv J., Guo S., Wei W. (2020). Heavy Oil Biodegradation by Mixed Bacterial Consortium of Biosurfactant-Producing and Heavy Oil-Degrading Bacteria. Pol. J. Environ. Stud..

[78] Zafra G., Taylor T.D., Absalon A.E., Cortes-Espinosa D.V. (2016). Comparative metagenomic analysis of PAH degradation in soil by a mixed microbial consortium. J. Hazard. Mater..

[79] Li X., Li P., Lin X., Zhang C., Li Q., Gong Z. (2008). Biodegradation of aged polycyclic aromatic hydrocarbons (PAHs) by microbial consortia in soil and slurry phases. J. Hazard. Mater..

[80] Wu M., Chen L., Tian Y., Ding Y., Dick W.A. (2013). Degradation of polycyclic aromatic hydrocarbons by microbial consortia enriched from three soils using two different culture media. Environ. Pollut..

[81] Bharali P., Bashir Y., Ray A., Dutta N., Mudoi P., Alemtoshi, Sorhie V., Vishwakarma V., Debnath P., Konwar B.K. (2022). Bioprospecting of indigenous biosurfactant-producing oleophilic bacteria for green remediation: An eco-sustainable approach for the management of petroleum contaminated soil. 3 Biotech..

[82] Reddy D.O., Milliken C.E., Foreman K., Fox J., Simpson W., Brigmon R.L. (2020). Bioremediation of Hexanoic Acid and Phenanthrene in Oil Sands Tailings by the Microbial Consortium BioTiger. Bull. Environ. Contam. Toxicol..

[83] Wang X., Chen J., Ji R., Liu Y., Su Y., Guo R. (2019). Degradation of Bisphenol S by a Bacterial Consortium Enriched from River Sediments. Bull. Environ. Contam. Toxicol..

[84] Zaborowska M., Wyszkowska J., Kucharski J. (2019). Biochemical activity of soil contaminated with BPS, bioaugmented with a mould fungi consortium and a bacteria consortium. Environ. Sci. Pollut. Res. Int..

[85] Golshan M., Jorfi S., Jaafarzadeh Haghighifard N., Takdastan A., Ghafari S., Rostami S., Ahmadi M. (2019). Development of salt-tolerant microbial consortium during the treatment of saline bisphenol A-containing wastewater: Removal mechanisms and microbial characterization. J. Water Process. Eng..

[86] Quintella C.M., Mata A.M.T., Lima L.C.P. (2019). Overview of bioremediation with technology assessment and emphasis on fungal bioremediation of oil contaminated soils. J. Environ. Manag..

[87] Mousavi S.M., Hashemi S.A., Iman Moezzi S.M., Ravan N., Gholami A., Lai C.W., Chiang W.H., Omidifar N., Yousefi K., Behbudi G. (2021). Recent Advances in Enzymes for the Bioremediation of Pollutants. Biochem. Res. Int..

[88] Zaborowska M., Wyszkowska J., Kucharski J. (2019). Soil enzyme response to bisphenol F contamination in the soil bioaugmented using bacterial and mould fungal consortium. Environ. Monit. Assess..

[89] Lu H., Weng Z., Wei H., Zhou J., Wang J., Liu G., Guo W. (2017). Simultaneous bisphenol F degradation, heterotrophic nitrification and aerobic denitrification by a bacterial consortium. J. Chem. Technol. Biotechnol..

[90] Inoue D., Hara S., Kashihara M., Murai Y., Danzl E., Sei K., Tsunoi S., Fujita M., Ike M. (2008). Degradation of Bis(4-Hydroxyphenyl)methane (bisphenol F) by *Sphingobium yanoikuyae* strain FM-2 isolated from river water. Appl. Environ. Microbiol..

[91] Sivasubramanian S., Namasivayam S.K.R. (2015). Phenol degradation studies using microbial consortium isolated from environmental sources. J. Environ. Chem. Eng..

[92] Nkanang A. (2020). Crude Oil Toxicity Tolerance of Hydrocarbonoclastic Strain of *Citrobacter Amalonaticus* -Y2esw1 Isolated from Estuarine Sediment in the Niger Delta of Nigeria. J. Microbiol. Biotechnol. Food Sci..

[93] Yuan X., Zhang X., Chen X., Kong D., Liu X., Shen S. (2018). Synergistic degradation of crude oil by indigenous bacterial consortium and exogenous fungus *Scedosporium boydii*. Bioresour. Technol..

[94] Shahebrahimi Y., Fazlali A., Motamedi H., Kord S., Mohammadi A.H. (2020). Effect of Various Isolated Microbial Consortiums on the Biodegradation Process of Precipitated Asphaltenes from Crude Oil. ACS Omega.

[95] Bidja Abena M.T., Sodbaatar N., Li T., Damdinsuren N., Choidash B., Zhong W. (2019). Crude Oil Biodegradation by Newly Isolated Bacterial Strains and Their Consortium Under Soil Microcosm Experiment. Appl. Biochem. Biotechnol..

[96] Tao K., Zhang X., Chen X., Liu X., Hu X., Yuan X. (2019). Response of soil bacterial community to bioaugmentation with a plant residue-immobilized bacterial consortium for crude oil removal. Chemosphere.

[97] Xu R., Zhang Z., Wang L., Yin N., Zhan X. (2018). Surfactant-enhanced biodegradation of crude oil by mixed bacterial consortium in contaminated soil. Environ. Sci. Pollut. Res. Int..

[98] Diallo M.M., Vural C., Cay H., Ozdemir G. (2021). Enhanced biodegradation of crude oil in soil by a developed bacterial consortium and indigenous plant growth promoting bacteria. J. Appl. Microbiol..

[99] Ebadi A., Khoshkholgh Sima N.A., Olamaee M., Hashemi M., Ghorbani Nasrabadi R. (2018). Remediation of saline soils contaminated with crude oil using the halophyte *Salicornia persica* in conjunction with hydrocarbon-degrading bacteria. J. Environ. Manag..

[100] Varjani S., Upasani V.N. (2019). Influence of abiotic factors, natural attenuation, bioaugmentation and nutrient supplementation on bioremediation of petroleum crude contaminated agricultural soil. J. Environ. Manag..

[101] Yi Z., Ma X., Song J., Yang X., Tang Q. (2019). Investigations in enhancement biodesulfurization of model compounds by ultrasound pre-oxidation. Ultrason. Sonochem..

[102] Chen W., Kong Y., Li J., Sun Y., Min J., Hu X. (2020). Enhanced biodegradation of crude oil by constructed bacterial consortium comprising salt-tolerant petroleum degraders and biosurfactant producers. Int. Biodeterior. Biodegrad..

[103] Huang X., Zhou H., Ni Q., Dai C., Chen C., Li Y., Zhang C. (2020). Biosurfactant-facilitated biodegradation of hydrophobic organic compounds in hydraulic fracturing flowback wastewater: A dose–effect analysis. Environ. Technol. Innov..

[104] Li H., Bao M., Li Y., Zhao L., King T., Xie Y. (2020). Effects of suspended particulate matter, surface oil layer thickness and surfactants on the formation and transport of oil-sediment aggregates (OSA). Int. Biodeterior. Biodegrad..

[105] Hu X., Qiao Y., Chen L.-Q., Du J.-F., Fu Y.-Y., Wu S., Huang L. (2019). Enhancement of solubilization and biodegradation of petroleum by biosurfactant from *Rhodococcus erythropolis* HX-2. Geomicrobiol. J..

[106] Wang C., Liu X., Guo J., Lv Y., Li Y. (2018). Biodegradation of marine oil spill residues using aboriginal bacterial consortium based on Penglai 19-3 oil spill accident, China. Ecotoxicol. Environ. Saf..

[107] Feng L., Jiang X., Huang Y., Wen D., Fu T., Fu R. (2021). Petroleum hydrocarbon-contaminated soil bioremediation assisted by isolated bacterial consortium and sophorolipid. Environ. Pollut..

[108] Ganesh Kumar A., Nivedha Rajan N., Kirubagaran R., Dharani G. (2019). Biodegradation of crude oil using self-immobilized hydrocarbonoclastic deep sea bacterial consortium. Mar. Pollut. Bull..

[109] Kamyabi A., Nouri H., Moghimi H. (2017). Synergistic Effect of Sarocladium sp. and Cryptococcus sp. Co-Culture on Crude Oil Biodegradation and Biosurfactant Production. Appl. Biochem. Biotechnol..

[110] Dehvari M., Ghafari S., Haghighifard N.J., Jorfi S. (2021). Petroleum Contaminated Seawater Detoxification in Microcosm by Halotolerant Consortium Isolated from Persian Gulf. Curr. Microbiol..

[111] Smulek W., Zdarta A., Luczak M., Krawczyk P., Jesionowski T., Kaczorek E. (2016). Sapindus saponins’ impact on hydrocarbon biodegradation by bacteria strains after short- and long-term contact with pollutant. Colloids Surf. B. Biointerfaces.

[112] Duan Y., Awasthi S.K., Liu T., Verma S., Wang Q., Chen H., Ren X., Zhang Z., Awasthi M.K. (2019). Positive impact of biochar alone and combined with bacterial consortium amendment on improvement of bacterial community during cow manure composting. Bioresour. Technol..

[113] Dai X., Lv J., Yan G., Chen C., Guo S., Fu P. (2020). Bioremediation of intertidal zones polluted by heavy oil spilling using immobilized laccase-bacteria consortium. Bioresour. Technol..

[114] Arpornpong N., Padungpol R., Khondee N., Tongcumpou C., Soonglerdsongpha S., Suttiponparnit K., Luepromchai E. (2020). Formulation of Bio-Based Washing Agent and Its Application for Removal of Petroleum Hydrocarbons From Drill Cuttings Before Bioremediation. Front. Bioeng. Biotechnol..

[115] Sridharan R., Krishnaswamy V.G., Kumar P.S. (2021). Analysis and microbial degradation of Low-Density Polyethylene (LDPE) in Winogradsky column. Environ. Res..

[116] Garcia-Sanchez M., Garcia-Romera I., Cajthaml T., Tlustos P., Szakova J. (2015). Changes in soil microbial community functionality and structure in a metal-polluted site: The effect of digestate and fly ash applications. J. Environ. Manag..

